# Prediction of Dry-Low Emission Gas Turbine Operating Range from Emission Concentration Using Semi-Supervised Learning

**DOI:** 10.3390/s23083863

**Published:** 2023-04-10

**Authors:** Mochammad Faqih, Madiah Binti Omar, Rosdiazli Ibrahim

**Affiliations:** 1 Department of Chemical Engineering, Universiti Teknologi PETRONAS, Seri Iskandar 32610, Malaysia; madiah.omar@utp.edu.my; 2 Department of Electrical and Electronics Engineering, Universiti Teknologi PETRONAS, Seri Iskandar 32610, Malaysia; rosdiazli@utp.edu.my

**Keywords:** Dry-Low Emission gas turbine, emission concentration, extreme gradient boosting, K-means, load management

## Abstract

Dry-Low Emission (DLE) technology significantly reduces the emissions from the gas turbine process by implementing the principle of lean pre-mixed combustion. The pre-mix ensures low nitrogen oxides (NO_x_) and carbon monoxide (CO) production by operating at a particular range using a tight control strategy. However, sudden disturbances and improper load planning may lead to frequent tripping due to frequency deviation and combustion instability. Therefore, this paper proposed a semi-supervised technique to predict the suitable operating range as a tripping prevention strategy and a guide for efficient load planning. The prediction technique is developed by hybridizing Extreme Gradient Boosting and K-Means algorithm using actual plant data. Based on the result, the proposed model can predict the combustion temperature, nitrogen oxides, and carbon monoxide concentration with an accuracy represented by R squared value of 0.9999, 0.9309, and 0.7109, which outperforms other algorithms such as decision tree, linear regression, support vector machine, and multilayer perceptron. Further, the model can identify DLE gas turbine operation regions and determine the optimum range the turbine can safely operate while maintaining lower emission production. The typical DLE gas turbine’s operating range can operate safely is found at 744.68 °C –829.64 °C. The proposed technique can be used as a preventive maintenance strategy in many applications involving tight operating range control in mitigating tripping issues. Furthermore, the findings significantly contribute to power generation fields for better control strategies to ensure the reliable operation of DLE gas turbines.

## 1. Introduction

Gas turbines are one of the most versatile and efficient power generation sources, used in various applications, including aviation, power plant, and oil and gas production. The primary operation in the gas turbine system is the combustion process, where the energy conversion takes place by mixing the compressed air and fuel, which is subsequently ignited to produce a high-temperature gas flow to rotate the turbine, producing a shaft work to drive the electrical generator. However, due to an incomplete reaction, combustion releases emissions, such as nitrogen oxides (NO_x_) and carbon monoxide (CO). The emission produced by combustion engines has become an topic in achieving net-zero emission target, which has positioned a stringent policy of pollution leading to the introduction of Dry-Low Emission (DLE) gas turbines [1].

**Figure 1:**
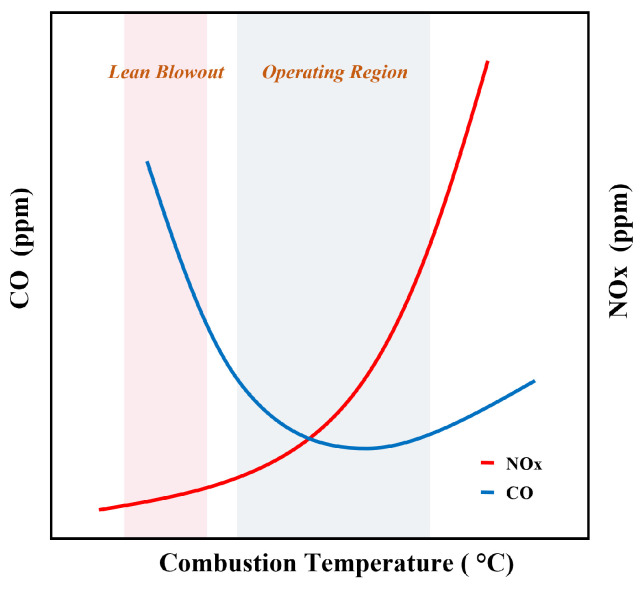
Illustration of the DLE gas turbine’s operating range [2].

DLE gas turbine reduces the emissions by implementing the lean-premixed (LPM) method to create a lower temperature by adding more air to be mixed with fuel before delivering it to the combustor, as lowering combustion temperature will lower the emission [2,3]. According to [4], the DLE gas turbine can achieve emission reduction up to 97%, which can effectively contribute to minimize the green house gasses. Even though this technology is environmentally friendly, the lean-burn operation may cause combustion instability due to various factors, including acoustic resonance and reduced flame speed, leading to flame-out, commonly known as lean blow-out (LBO) [5]. LBO fault exists when the turbine operates below the LBO limit, the lowest equivalence ratio that can carry on the flame [6]. In addition, combustion at too low a temperature leads to the high formation of CO emissions. On the other hand, operating the turbine higher than the desired range will lead to high emission production of NO_x_ [7,8]. Therefore, the DLE gas turbine operation should be maintained in a specific range, as illustrated in Figure 1.

Various causes of LBO are identified, such as frequency fluctuation and unbalanced air fuel ratio due to sudden change of load, as rapid demand on load affects the combustion stability of DLE gas turbine [9,10]. When the load decreases, the fuel flow rate decreases, leading to a leaner air-to-fuel ratio. This leaner mixture can cause the flame to become unstable and eventually lead to a lean blowout [11]. Similarly, when the load on the gas turbine increases, the fuel flow rate increases, leading to a richer mixture. This richer mixture can also lead to instability in the flame due to incomplete combustion. Therefore, proper load management is essential in maintaining the healthy operation of the DLE gas turbine, which can be achieved by carefully determining the operating range.

According to Figure 1, the operating range is a function of NO_x_ and CO emissions against the combustion temperature. Hence, the suitable operating range can be estimated by predicting the emission of NO_x_ and CO produced during the combustion process. Numerous prediction of emission predictions is available in the literature. A numerical model was proposed by Emami in [12] to predict the NO_x_ and CO concentration using Computational Fluid Dynamics (CFD). The numerical simulation was used to identify the mechanism of NO_x_ formation and CO characteristics concerning the change in inlet air temperature. The other method uses semi-empirical analysis by combining CFD and Chemical Reactor Network (CRN) to predict the emission composition and LBO event [13]. Another study from [14] implemented a statistical method by employing response surface methodology-based Box–Behnken design to model and optimize the prediction of NO_x_ and CO emissions from diesel engine. These approaches demonstrated a good result. However, there is a lack of physical insights since the prediction was made using a numerical simulation underlying the physical model.

The data-driven method is subsequently adopted because it can predict the result by only learning from data. Hence, it simplifies the calculation and reduces the possibility of a lack of physical law during model development. Three approaches are commonly used in data-driven methods: supervised, unsupervised, and semi-supervised. In emission prediction, supervised learning is mainly implemented to predict the amount of concentration. Masoud [15] and Saiful [16] implemented Support Vector Machine (SVM) regressor to predict NO_x_ for diesel engines and gas turbines, respectively. Tuttle further combined SVM with Neural Networks (NNs) to classify and predict the emission from different fuels [17]. Bo Liu [18] also employed SVM by combining the model with Principal Component Analysis (PCA) and Genetic Algorithm (GA) to predict NO_x_ concentration, which outperforms other algorithms such as the original SVM, neural networks, and Partial Least Squares (PLS). On the other hand, a non-parametric supervised method, namely k-nearest neighbors (k-NN), is used by Rezazadeh to predict NO_x_ [19]. Meanwhile, other scholars prefer implementing NNs algorithms, such as Botros [20] and Minxing [21], who employed NNs to predict NO_x_ from the conventional and DLE gas turbine, respectively. NNs-based prediction has also found promising results for many applications, such as for forced convection and thermal predictions [22].

A challenge encountered in dealing with gas turbine data is the existence of noisy and missing data due to the heavy operation of the turbine. Hence, selecting a proper prediction technique is necessary to develop an adaptive model that can handle the corrupted data correctly and efficiently. An ensemble algorithm, namely extreme gradient boosting (XGBoost), has the capability to manage these matters as established by Minxing Si in [23], which successfully outperforms the neural networks model in predicting the NO_x_ emission from a coal-fired boiler. Therefore, XGBoost is employed in this study for emission prediction due to effortless data preprocessing, less time model training, and fewer hyperparameters to adjust. In addition, this algorithm can also handle large datasets and has achieved state-of-the-art performance on many prediction method benchmark.

Despite the advantages of XGBoost for predictive modeling tasks, the supervised approach may not be suitable for determining the operating range since it is limited to labeled data and supervision only. The operating range of the gas turbine is affected by various factors, such as the fluctuation of the ambient conditions and the operation demand, creating ambiguity in defining the exact range. Therefore, the clustering approach is adopted in this study to predict the operating range by discovering the similarity in the data and grouping them in distinct regions. K-means is one of the most widely used algorithms in the clustering approach, which is categorized as a partitioning method [24,25]. K-Means is a well-known unsupervised learning-based algorithm introduced 50 years ago [26] and favored due to fast computation, simplicity, and ability to handle huge data [27]. In addition, it is suitable for dealing with unevenly distributed data and producing consistent results with different initializations [28].

Therefore, this paper aims to propose a prediction technique to determine the DLE gas turbine’s operating range based on the emission concentration by hybridizing XGBoost and K-Means algorithms. The main contributions of the paper are highlighted as follows:

- Develop a model to predict the emission of NO_x_ and CO from DLE gas turbine using XGBoost. Additionally, the combustion temperature will be predicted.
- Develop a technique to predict the operating range of a DLE gas turbine based on gas emission concentration using K-means algorithm.

Furthermore, several data-driven techniques based on machine learning methods will also be employed to study different applications of the algorithms and provide an overview of their prediction capability in the studied case. The proposed hybrid model contributes to the development of emission reduction of power generation while proving a healthy operation during the DLE mode. Additionally, the proposed technique is adaptable for other implementations involving engine operation that require an operating range control strategy.

## 2. Semi-Supervised Learning for Operating Range Prediction of Dry-Low Emission Gas Turbine

**Figure 2:**
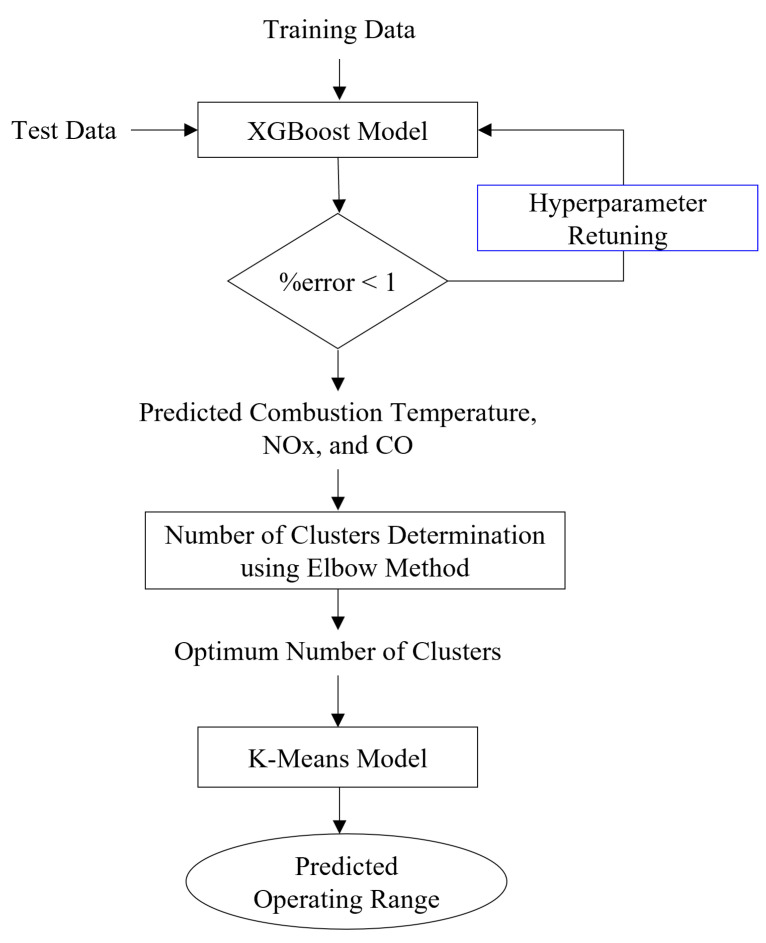
Flowchart of model development.

This section is divided into two subsections presenting the XGBoost and K-Means algorithm description. The overall flow of the model development is depicted in Figure 2. Firstly, the data of a DLE gas turbine collected from the actual plant are divided into training and test data with a division ratio of 70:30. The training data are further carried out pre-processing using the Pearson correlation test to determine the important features for model input. In addition, a technical description for each parameter impacting the turbine operation will also be evaluated to ensure the feature selection. Subsequently, the data are trained to develop the regression model of XGBoost predicting the combustion temperature, NO_x_, and CO emissions. After that, the test data are fed to predict the new result and validate the model performance. In this part, the developed model will be compared with other regression algorithms, such as decision tree, linear regression, multilayer perceptron, and support vector machine. The predicted output is then used for operating range prediction. The next step is to determine the cluster using the elbow method. The optimum cluster is further taken for K-means model development. Lastly, the predicted region is assigned based on the operating condition of the DLE gas turbine to find the optimum range in which the turbine can operate.

### 2.1. XGBoost

**Figure 3:**
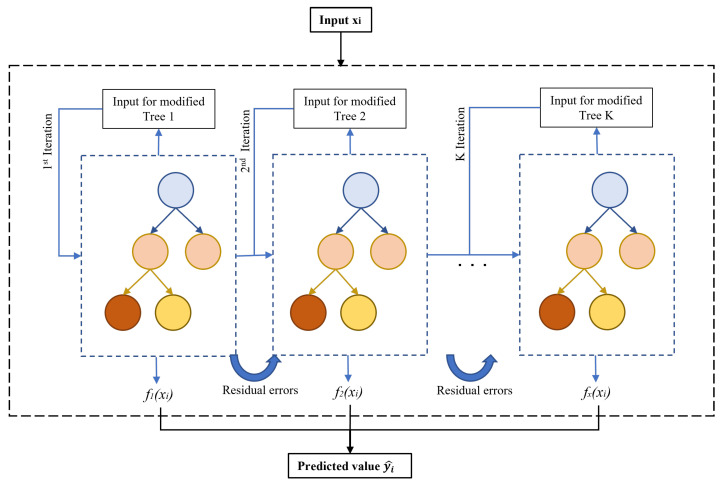
XGBoost approach concept.

Extreme gradient boosting (XGBoost) algorithm is a tree-based ensemble learning that first time released in 2014. The idea of XGBoost comes from boosting method that is expressed as:

(1) $${\hat{y_{i}}}^{k}={\hat{y_{i}}}^{k-1}+f_{k}(x_{i})$$

where ${\hat{y_{i}}}^{k}$ is the predicted output for ith data and *k* is the number of iterations. $f_{k}\left(x_{i}\right)$ is the estimator to improve the previous prediction ${\hat{y_{i}}}^{k-1}$. The architecture of tree-based learning is illustrated in Figure 3, where it includes root nodes as represented by blue circles, internal nodes with faded orange circles, and leaf nodes with brown and yellow circles.

In XGBoost, a regularization function is introduced to avoid overfitting and optimizes the loss function. The objective function or loss function for regression problem is expressed as:

(2) $$J=\sum_{i=0}^{n}L\left(y_{i},\hat{y_{i}}\right)+\sum_{k=0}^{n}\Omega \left(f_{k}\right)$$

where *n* denoted as the number of training samples and $\Omega (f_{k})$ is a regularization function. The regularization function is written as:

(3) $$\Omega \left(f_{k}\right)=\gamma T+0.5\lambda \sum_{j=0}^{T}w_{j}^{2}$$

where *T* is the number of leaf nodes and *w* is the leaf weight. γ and λ are the hyperparameters that can be tuned to improve the performance and produce a great prediction result. The training process is repeated iteratively, with new trees being added that forecast the residuals or errors of previous trees, which are then integrated with previous trees to provide the final prediction.

In order to improve the performance of the proposed model, a hyperparameter tuning of XGBoost is employed. The optimization is performed based on cross validation with cv value of 5. Some parameters which potentially gives a better contribution are also manually tuned. The finalized hyperparameters used for model development are tabulated in Table 1.

The predicted value from the model will be evaluated against the actual value using three performance parameters, which are $R^{2}$, mean absolute error (MAE), root mean squared error (RMSE), and relative percentage error (%error), expressed as follows; where the $\hat{y}$ is the predicted value of *y*.

(4) $$R^{2}=1-\frac{\sum_{i}{\left(y_{i}-\hat{y_{i}}\right)}^{2}}{\sum_{i}{\left(y_{i}-\bar{y}\right)}^{2}}$$

(5) $$MAE=\frac{1}{n}\sum_{i=1}^{n}\left|y-\hat{y}\right|$$

(6) $$RMSE=\sqrt{\frac{1}{n}\sum_{i=1}^{n}{\left(y-\hat{y}\right)}^{2}}$$

(7) $$\%\;error=\frac{|Actual\;Value\left(y\right)-Predicted\;Value\left(\hat{y}\right)|}{Actual\;Value(y)}\times 100\%$$

### 2.2. K-Means

**Figure 4:**
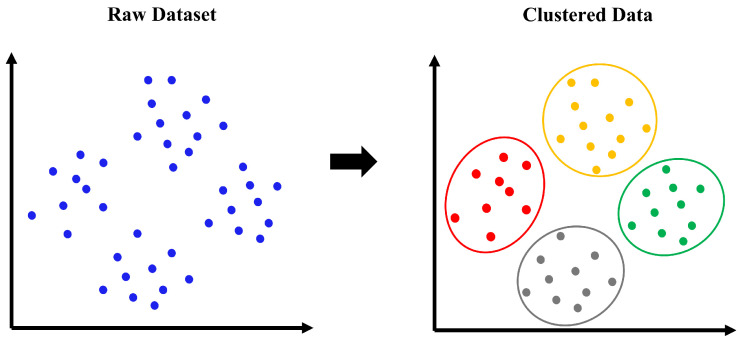
K-means concept.

K-means clustering has been proved its convergence for many years ago, opening the way for its widespread application in current research and industry [29]. The approach involves selecting *k* randomly as the initial clustering center, calculating the distance between each object and the initial clustering center, and assigning it to the nearest clustering center [30]. The clustering center as known as centroid and the items that have been assigned to them represent a group of classes, as represented by different colours of data groups in Figure 4. The cluster center will be recalculated for each object assigned according to the the cluster’s existing items. The loop continues until the cluster center is no longer changing.

The K-means method is divided into two steps. The first step is determining the initial *k*. In this research, the elbow method is selected to find the proper value of the initial *k*. The *k* range used in this study varies from 2 to 10 and is then plotted against the WCSS (within-cluster sum of square), also known as inertia, which is calculated by summing the squared distance between each point and the centroid in a cluster. The value of inertia will decrease as the cluster increases. At the point when the inertia starts to move almost parallel to the X-axis is the elbow point, where the optimum value of *k* is found.

The second step is determining where each object belongs in the cluster. In this stage, the Euclidean distance is calculated for ith object $o_{i}$. The Euclidean value represents the distance between $o_{i}$ and each of the cluster-centers $k_{j}$. Subsequently, we must observe the corresponding cluster center $S_{j}$ with the smallest distance. The calculation is formulated by Equation (8),

(8) $$d_{\left(o_{i},j_{k}\right)}=\sqrt{\sum_{m=1}^{M}{\left(o_{i,m}-k_{j,m}\right)}^{2}}$$

where *M* is the total number of features, $o_{i}$, *m* is the value of the $m_{th}$ feature of the $i_{th}$ object, and $k_{j,m}$ is the value of the $m_{th}$ feature of the $j_{th}$ cluster center.

## 3. Data Collection and Preprocessing

### 3.1. Data Collection

The data were collected from 4 months of a DLE gas turbine operation, which consists of 100,000 data points on healthy and unhealthy conditions. The healthy operation represents the data that were collected during the normal operation. The unhealthy data contain three incidents of trips, implying the information on the undesirable operation that happened during the data-collection period. The gas turbine type is a two-stage single shaft with rated power of 17.9 MW. The turbine has 16 stages axial flow compressor and fueled by natural gas.

The data consist of 13 operating parameters and 2 emission parameters of the DLE gas turbine, as tabulated in Table 2. The operational parameters consists of load, speed, ambient air temperature, inlet guide vane opening, compressor discharge pressure, stop ratio valve opening, gas control valve opening, splitter opening, fuel gas flow, fuel gas pressure, T5 combustion temperature, T7 exhaust temperature, and exhaust gas pressure. The gas emissions measured by the gas analyzer are NO_x_ and CO concentration.

**Figure 5:**
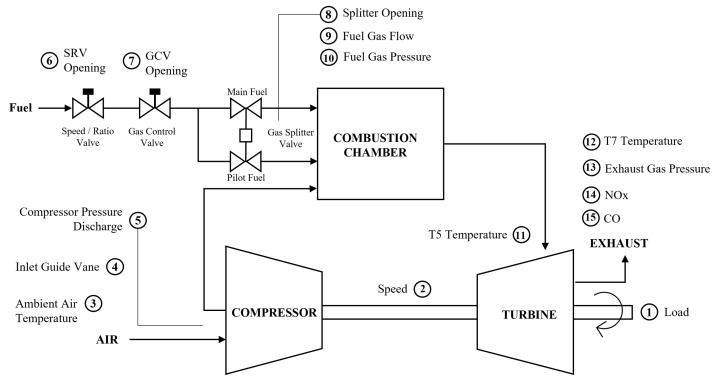
System flow diagram of the DLE gas turbine.

Figure 5 illustrates the system flow diagram of the typical DLE gas turbine with the measurement sensors. Three main components of the gas turbine arrangement are observed by sensors: compressor, combustion chamber, and mechanical turbine. The load demand maneuvers the gas turbine operation, as mentioned in 1. The driven load determines the power output by maintaining the rotation of the mechanical turbine at a certain speed in 2. The power output is sensitive to the ambient air temperature, which refers to 3 as an increase in ambient air temperature lowers the air density, reducing the mass flow through the turbine, and decreasing the power output. Hence, monitoring the air ambient temperature is essential in maintaining the reliable operation and performance of the gas turbine. The air is directed to the compressor by IGV at 4, and then compressed with pressure discharge monitored as CDP at 5 before mixing with the fuel in the combustion chamber. The fuel coming to the combustion chamber enters SRV as mentioned in 6 to maintain the gas pressure stable and regulate the pressure drop. The GCV at 7 then regulates the fuel flow as required for the combustion process. Since the DLE combustor type requires two partitions of fuel, the splitter valve, as mentioned in 8, controls the splitting of the main fuel and pilot fuel before entering the chamber. The flow and pressure of the fuel are monitored at 9 and 10, respectively.

The combustion temperature is difficult to monitor due to the extreme conditions and thermal gradient inside the chamber. The firing temperature is proportional to the gas temperature leaving the chamber. Hence, the temperature is measured at the exhaust of the chamber as labeled by T5 at 11, as measuring the temperature in the combustion chamber is not possible due to physical sensor limitations. Therefore, T5 is considered the combustion temperature in this study, which will be used for operating range prediction. In the exhaust part of the turbine, the temperature and pressure are monitored as 12 and 13. The emission of NO_x_ and CO produced during the process is measured at 14 and 15, respectively.

#### Data Analysis

**Figure 6:**
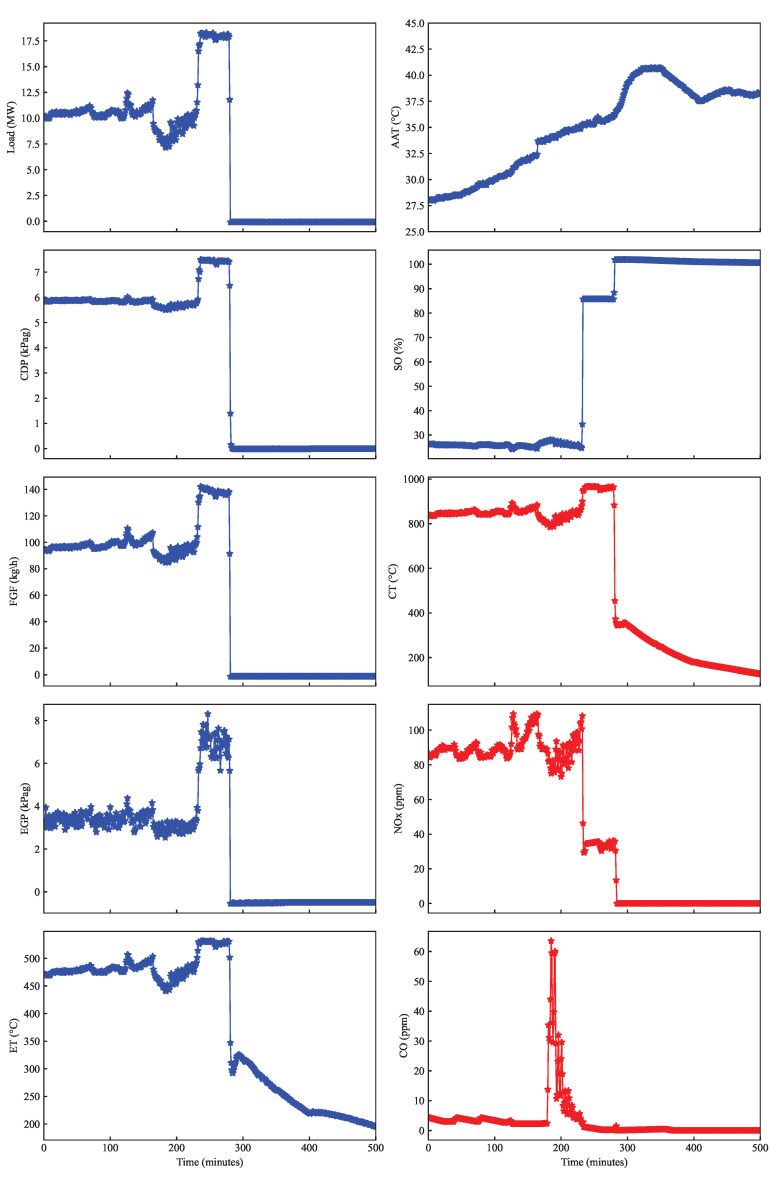
Trip signature from parameters of the DLE gas turbine.

A sample data collected from the typical DLE gas turbine are captured in Figure 6, where the input and output parameters are represented by blue and red lines, respectively. The data contain a trip incident after 280 min, as indicated by the load going down suddenly to 0 MW. Before the trip occurred, there was a sudden increase in load from 10 MW to 18 MW at 238 min. The system maintained the desired load for several minutes before it went failed, then the load significantly dropped.

Further analysis exhibits a similar trend of CDP and FGF, where both parameters rise quickly due to the sudden load increase. Since the gas turbine is a single-shaft type with relatively constant speed, the increase in load demand is followed by an increase in the fuel flow or FGF, which raises the combustion temperature, CT. Thus, a large opening of the splitter, SO, is identified from 25% to 88% during load change; then, it increased to 100% or was fully open during the trip. The increase in CT affects the rise of CDP, where CDP is used to estimate the firing temperature reference. Other parameters, such as exhaust temperature (ET) and exhaust gas pressure (EGP), also have an identical pattern in which the value rises at a top point before the trip occurrence. With closer observation, the ambient temperature, AAT, increased gradually during the trip up to 40 °C, revealing a fluctuation in ambient conditions. Similarly, the concentration of NO_x_emission fluctuated before the trip happened. On the other hand, CO emissions significantly increased from 2 ppm to 60 ppm before the trip occurred.

By carefully observing the phenomenon of sudden load increase in DLE gas turbine, it can be examined that the transient condition may cause dynamic instability leading the turbine to trip. In addition, due to rigorous ambient and operational settings, the root cause of the tripping incident might be difficult to recognize. The unsupervised learning can identify the patterns and structure in the data independently and even uncover hidden relationships by grouping the data based on its similarity. Therefore, implementing unsupervised learning such as K-means will help discover the operating region of DLE gas turbine in which the data contain healthy and unhealthy operations. It allows the engineers to identify different operating regimes or conditions that the gas turbine may be operating in. This information can then be used to optimize the gas turbine’s performance for each of these operating conditions, leading to improved efficiency and reduced maintenance costs. Similarly, this approach can be implemented to other applications of engines with noisy or incomplete operational data to identify the anomalies and reveal hidden relationships and insights that may be difficult to detect through manual analysis.

### 3.2. Data Pre-Processing

**Figure 7:**
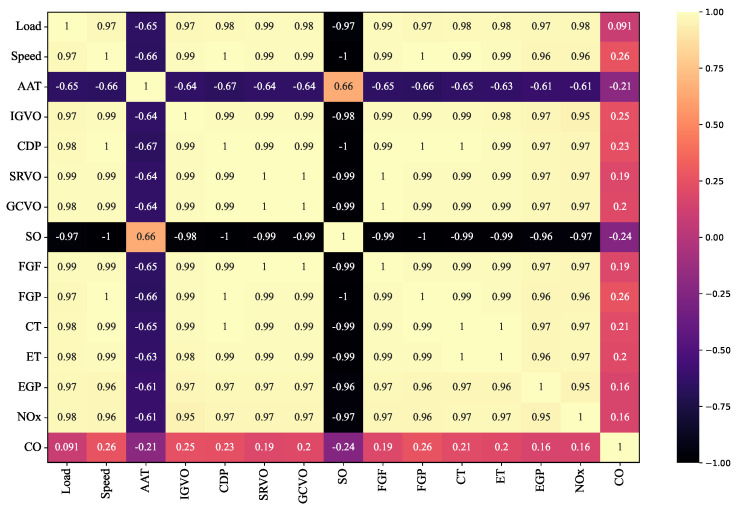
Parameters correlation test.

In order to perceive the relationship between the parameters of the dataset, the correlation test result is mapped in Figure 7. The relationship is then analyzed based on the correlation of each input parameter and the pairwise correlation between input and target parameters. The correlation test is performed by calculating Pearson’s correlation as described by Equation (9).

(9) $$r=\frac{N\sum (xy)-(\sum x)(\sum y)}{\sqrt{\left[N\sum x^{2}-{\left(\sum x\right)}^{2}\right]\left[N\sum y^{2}-{\left(\sum y\right)}^{2}\right]}}$$

where *N* is the number of pairs of scores, ∑(xy) is the sum of the products of paired scores, ∑(x) and ∑(y) are the sum of *x* and *y* scores, and $\sum (x^{2})$ and $\sum (y^{2}$) are the sum of squared *x* and *y* scores.

Firstly, by carefully observing the correlation of input parameters, the highest correlated parameter is CDP, followed by FGP, Speed, SO, and FGF. CDP has a strong correlation value of 1 with speed and FGP. However, speed and FGP are not employed since the typical turbine is a single-shaft type in which the engine has to operate at a relatively constant speed, and the gas pressure is maintained at a particular value. Meanwhile, CDP hugely contributes to estimating the turbine inlet’s temperature reference. Thus, CDP is preferred to speed and FGP. Load is also highly correlated to other parameters, with the highest correlation value of 0.99 against FGF. The role of the load in maneuvering the turbine operation has positioned this parameter as an essential feature to be considered for model development. Further, various operating conditions can be affected by the fluctuation of the load, making this parameter more necessary to be examined. Other highly correlated parameters, SO and FGF, have a high correlation value of 0.9 against other parameters. These two parameters significantly impact the DLE gas turbine system since the output power is adjusted by regulating the FGF, which can be controlled through the SO. Therefore, SO and FGF are essential for model development representing the gas fuel system. In the exhaust component, ET and EGT have the highest correlation with other input parameters, with a correlation value above 0.95, except with AAT. Nevertheless, AAT significantly impacts gas turbine performance since the fluctuation in it will affect the output power.

Secondly, the correlation between input and target variables exhibits a high dependency, as summarized in Table 3. CDP and ET have the highest correlation against the combustion temperature, CT, with a correlation value of 1. The other parameters also correlate highly with CT, with an average correlation of 0.9. Examined from the emission predicted target, NO_x_ gains a higher correlation value than CO for all input parameters, with the highest correlation from load (0.98) and the lowest being AAT with a correlation value of −0.61. CO emission is highly correlated with FGP and speed, with a correlation value of 0.26. Other parameters also portray a relatively high correlation, except the load, which is 0.091. Nevertheless, the load is considered an essential parameter since the load demand drives the gas turbine operation. Therefore, based on the correlation analysis, the finalized parameters used for model development are CDP, SO, FGF, EGP, ET, Load, and AAT.

## 4. Results and Discussion

This section discusses the results of the predicted combustion temperature, NO_x_, and CO emissions from the proposed XGBoost model. Furthermore, the comparison of the proposed model and other algorithms is also presented. Subsequently, the prediction result of DLE gas turbine operating range from a K-means model is discussed.

### 4.1. Prediction of Combustion Temperature, NO_x_, and CO Using XGBoost

The regression model has been developed using XGBoost to predict three output parameters: combustion temperature (CT), NO_x_, and CO. The result is analyzed based on the graphical plot and numerical evaluation. A benchmark of the proposed model against other algorithms is also discussed, as summarized in Table 4.

#### 4.1.1. Combustion Temperature Prediction

**Figure 8:**
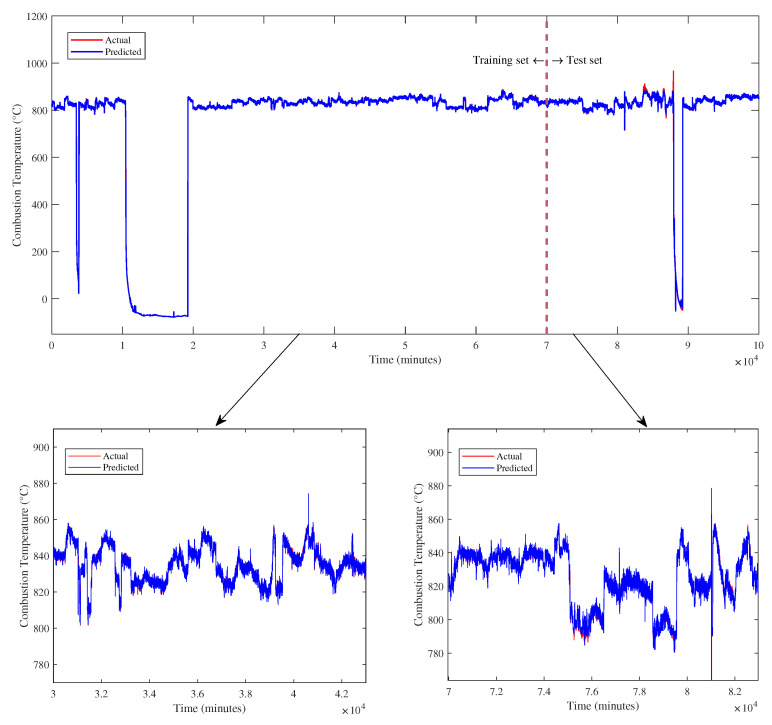
Combustion Temperature prediction from the proposed model.

Figure 8 presents the plot of combustion temperature (CT) prediction for the training and test dataset. In the figure, it can be examined that the model successfully predicts the test data capturing the trend when the trip happens as the temperature goes down to 0 °C and during start-up until it reaches the desired temperature at normal operation.

Based on the numerical evaluation of performance metrics, the proposed model performs excellently by obtaining an $R^{2}$ of 0.9999, MAE of 1.1285, RMSE of 6.9549, and %error of 0.0356. The MAE of the XGBoost model is the third lowest after linear regression and support vector machine. On the other hand, the RMSE and relative error percentage (%error) are the second largest after decision tree. The model of CT prediction is acceptable since the relative error percentage meets the decision criteria, which is less than 1%. Even though the error metrics of the proposed model are not the lowest among other algorithms, it still exhibits a promising result as the errors are very few.

Based on the graphical evaluation, it can be seen from the bottom right of Figure 8 that the actual and predicted CT values are very coincident, indicating that the predictions can follow the actual values precisely. Furthermore, the predicted training data also converge to the actual data, indicating well-fitted data, as shown in the bottom left of the figure.

**Figure 9:**
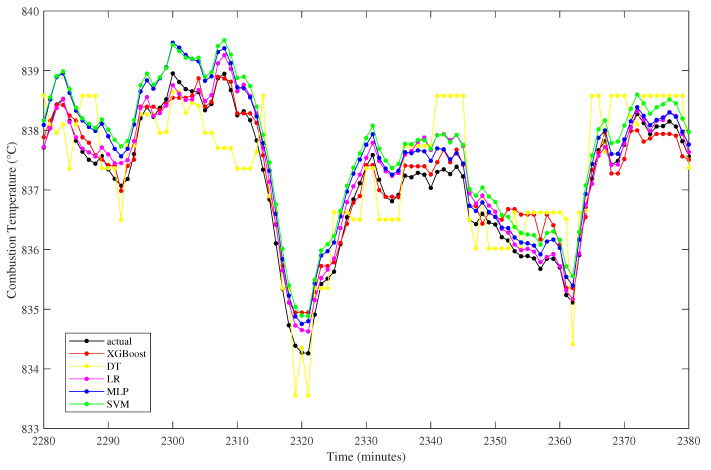
Combustion Temperature prediction from different algorithms.

The proposed model is further compared graphically with other algorithms, as depicted by the zoomed plot of the predicted test data in Figure 9. Based on the figure, it can be examined that the proposed XGBoost and linear regression have the closest line to the actual one. The support vector machine and multilayer perceptron are slightly distant from the actual one, while the decision tree model has fluctuating predicted values. The fluctuation of the decision model shows a higher deviation than others, as confirmed by the highest RMSE gained, which is 7.3604.

**Figure 10:**
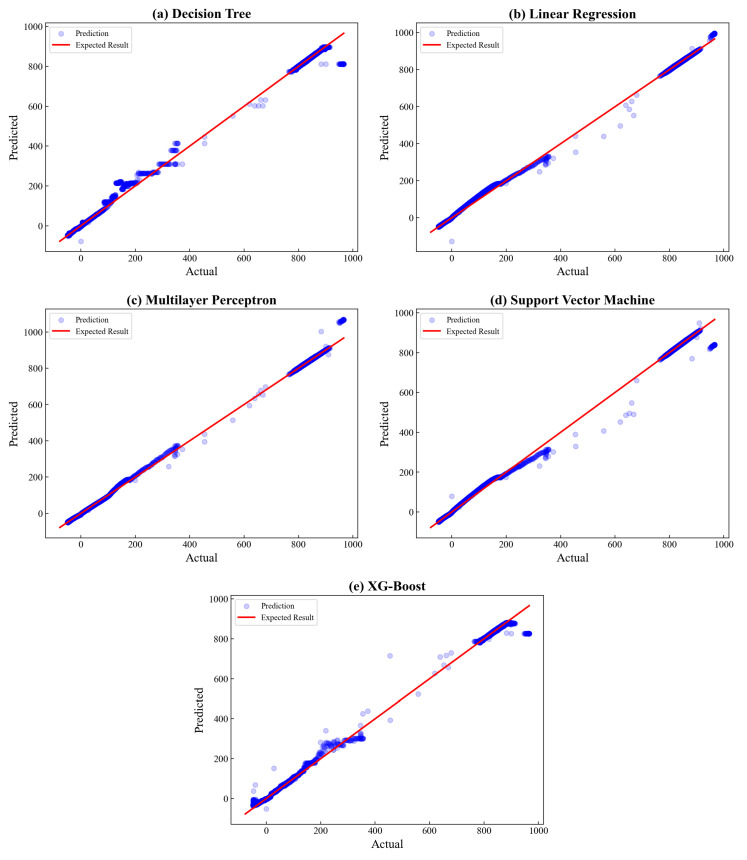
Actual vs. predicted plot of Combustion Temperature.

The goodness of fit between actual and predicted values is visualized in Figure 10, where the blue represents the data points scattered, and the red line depicts the expected results. Based on the visualization, it can be seen that all algorithms exhibit a well-fitted result as the data points are nearly evenly distributed to the expected result. Furthermore, only some data points are placed distant from the expected result. Therefore, it can be concluded that all the algorithms produce promising results for CT prediction. Nevertheless, the proposed model of XGBoost gains the highest $R^{2}$ value, which is 0.9999, revealing the best fit among others, followed by linear regression (0.9996) and multilayer perceptron (0.9993).

#### 4.1.2. NO_x_ Prediction

**Figure 11:**
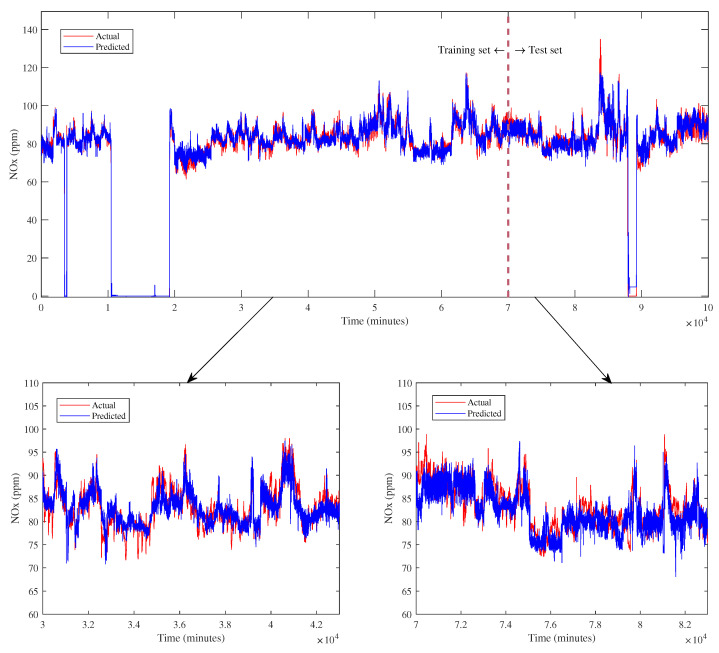
NO_x_ prediction from the proposed model.

The proposed model exposes an excellent result of NO_x_ prediction as depicted in Figure 11. According to that figure, the proposed model effectively predicts the actual data of the trend during the trip incident and normal operation for both the training and test data sets.

The numerical analysis exhibits that the proposed XGBoost model can outperform other algorithms by raising the highest $R^{2}$ and the lowest RMSE, which are 0.9309 and 4.9765, respectively. However, the proposed model gains the second lowest MAE after multilayer perceptron with a slightly different; XGBoost (3.5968) and multilayer perceptron (3.2349). Nevertheless, the proposed model meets the decision criteria by reaching the relative error percentage of 0.1168, which is the lowest among other algorithms.

With closer observation based on graphical analysis, the predicted trend of NO_x_ emission can follow the actual one as represented by red and blue lines for both the training and test data sets. This result indicates that the proposed model is capable of capturing the NO_x_ emission, which fluctuated due to complex chemical processes during the combustion.

**Figure 12:**
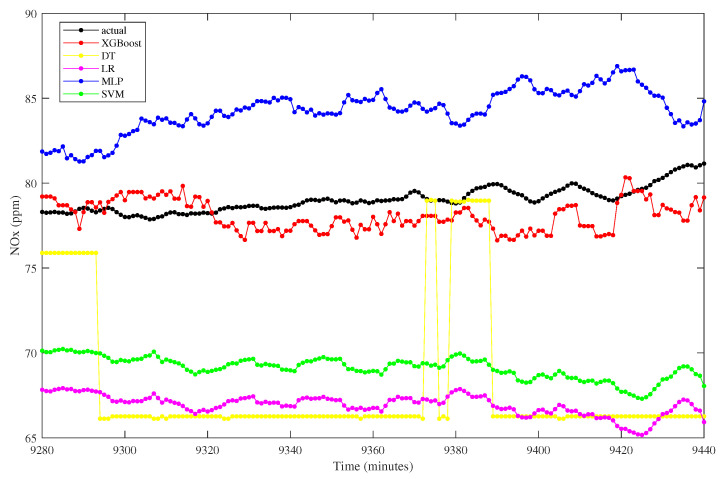
NO_x_ prediction from different algorithms.

The prediction from all algorithms is depicted in Figure 12. It can be graphically examined that the proposed model has a close line to the actual one. On the other hand, the predicted values of linear regression and support vector machine fall slightly away from the actual trend. Similarly, the decision tree model also has a distant trend against the actual one, with fluctuated values at some points. The poor linear regression prediction can be explained numerically by gaining the highest RMSE than others, which is 8.7526. Similarly, the decision tree and support vector machine have the second and third highest RMSE. Furthermore, the deviation of the decision tree model is represented by the relative error percentage of 7.2016, which is the highest among the others.

**Figure 13:**
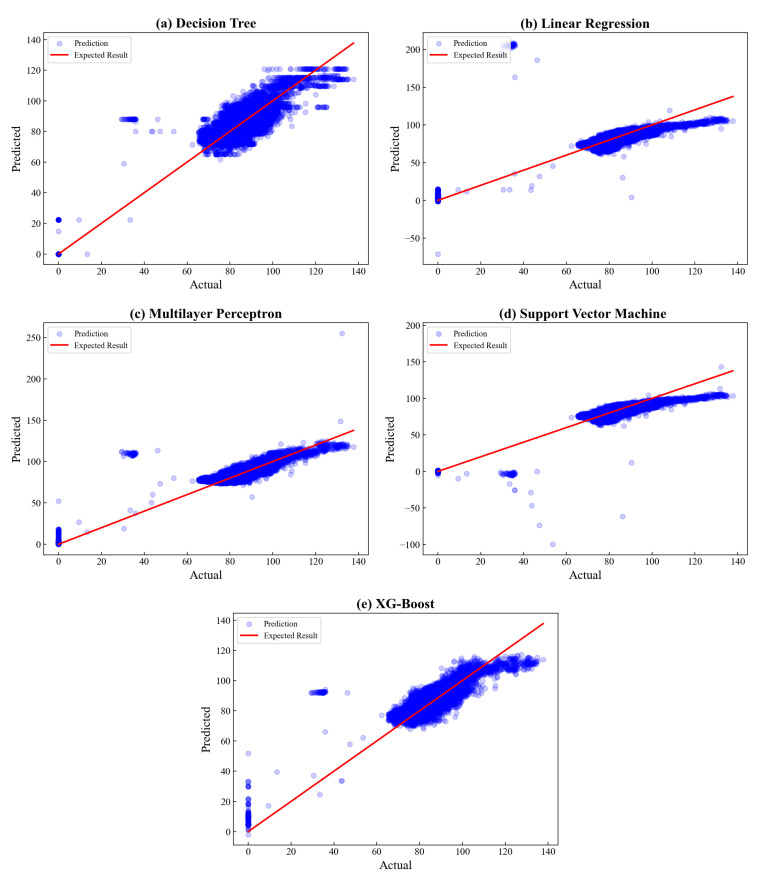
Actual vs. predicted plot of NO_x_.

Further analysis is carried out by plotting the actual and predicted values as visualized in Figure 13. Based on the plot, the data are mainly distributed at 60–137.69 ppm, showing the amount of NO_x_ concentration emitted during the operation. By careful observation, the proposed XGBoost yields a proportional plot with fewer data points which fall away from the expected result compared to the others. This result exhibits a well-fitted prediction, which can be described by raising the highest fitness coefficient of $R^{2}$ = 0.9309. In contrast, the linear regression model has more distant data points against the expected result, representing poor prediction. This result can be explained numerically by gaining the lowest $R^{2}$ value, which is 0.8008.

#### 4.1.3. CO Prediction

**Figure 14:**
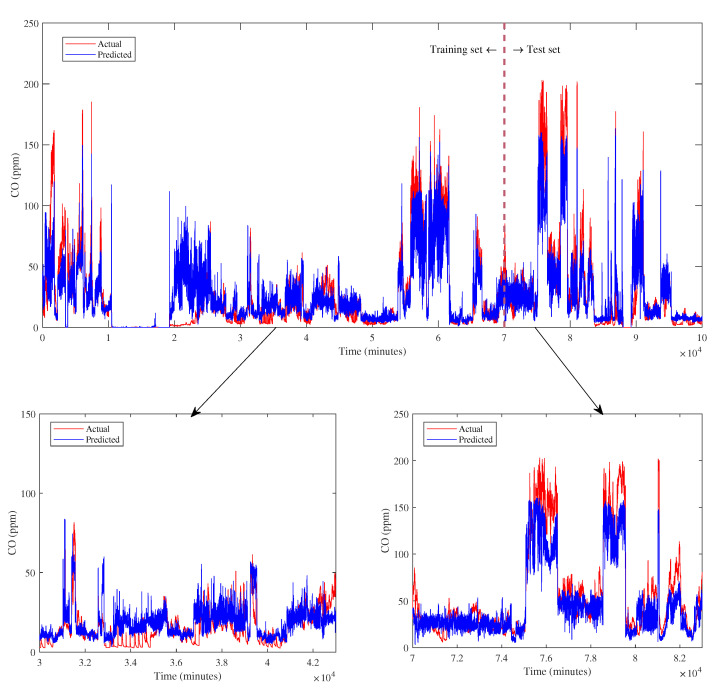
CO prediction.

The prediction of CO emission from the proposed model exhibits a promising result, as depicted in Figure 14. The trend during normal operation and trip occurrence is successfully predicted for training and test data sets.

Based on the numerical evaluation of performance metrics, the proposed XGBoost model outperforms other algorithms by obtaining the highest $R^{2}$ of 0.7109 and the lowest RMSE of 23.7489. However, the MAE of the proposed model is the second lowest after multilayer perceptron; XGBoost = 3.5968 and multilayer perceptron = 3.2349. Nevertheless, the proposed XGBoost model gains the lowest relative error percentage, which is 0.9200, showing that the model meets the decision criteria. Even though XGBoost capably predicts CO emission, the performance is lower than other predicted outputs, such as combustion temperature and NO_x_. This result can be estimated by examining the correlation value of CO against features that are lower than NO_x_ and combustion temperature.

**Figure 15:**
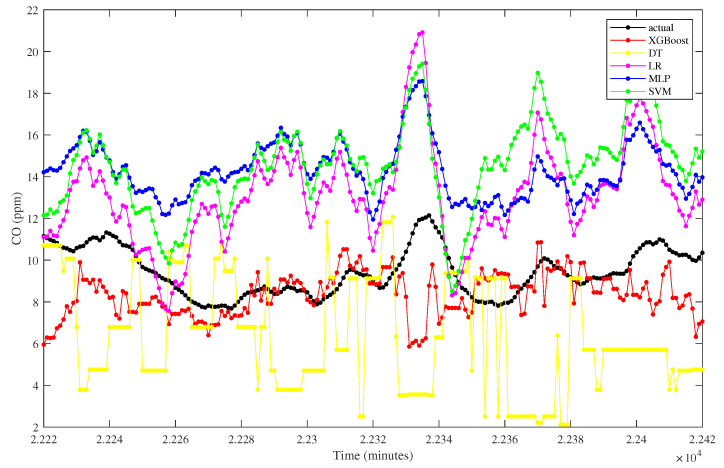
CO prediction comparison.

The comparison of CO prediction from the proposed model and other algorithms is visualized in Figure 15. Based on the plot, it can be examined that the XGBoost model has a closer trend with the actual one compared to others, revealing an excellent prediction result. This result can be explained by the relative error percentage in which the XGBoost model gains the lowest error. In contrast, the decision tree has fluctuated predicted values as represented by a significant deviation on the plot.

**Figure 16:**
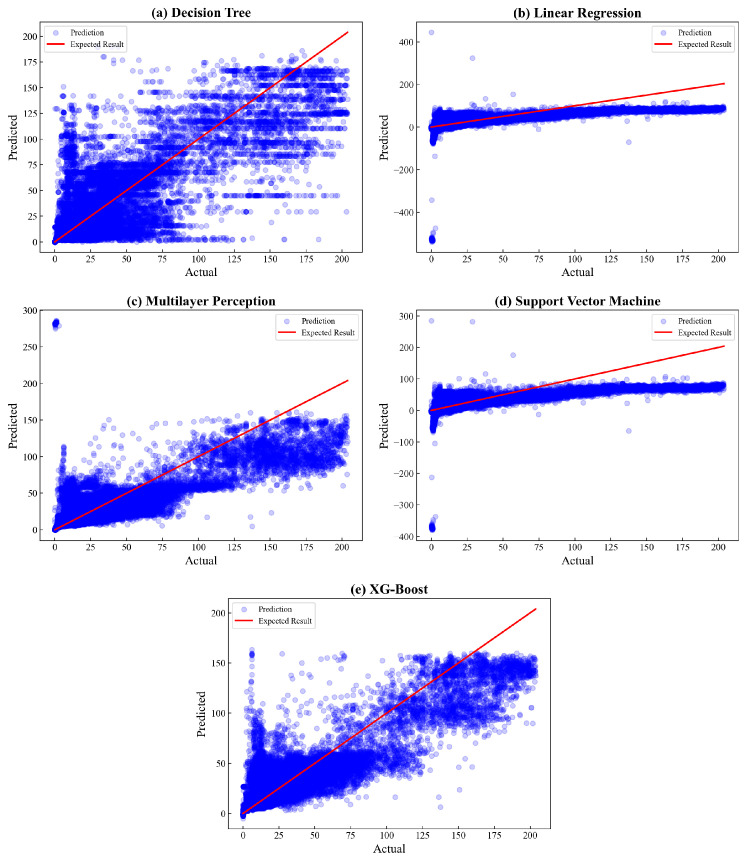
Actual vs. predicted plot of CO comparison.

The fitness between actual and predicted values is depicted in Figure 16. Based on the plot, it can be seen that the XGBoost model outperforms other algorithms, as shown by the evenly distributed data being spread closer to the expected result than others. In contrast, linear regression, SVM, and MLP have several data points too far from the expected result, indicating a huge deviation. These results can be numerically explained by the rank of $R^{2}$ value in which the proposed model gains the highest coefficient of 0.7109, followed by MLP with a value of 0.6918.

### 4.2. Clustering Model Development in K-Means

**Figure 17:**
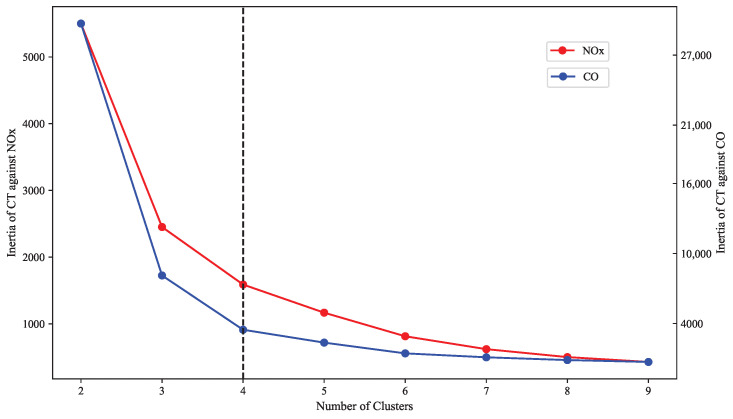
Cluster number determination using elbow method.

The predicted values of combustion temperature, NO_x_, and CO emissions from the XGBoost model are subsequently used to predict the DLE gas turbine’s operating range. The prediction starts by determining the number of clusters using Elbow method, as shown in Figure 17.

The inertia is a function of the number of clusters in which the point where its rate starts decreasing to level off is considered the optimal number of clusters. The inertia calculation is carried out separately for NO_x_ and CO emissions against the combustion temperature for more accurate results. Additionally, it will provide an easier visualization to determine the elbow point where the optimal cluster is found. Based on the elbow plot, it can be seen that both the inertia rate of NO_x_ and CO starts decreasing at cluster number 4. Hence, the predicted data will be clustered into four distinct operating regions.

**Figure 18:**
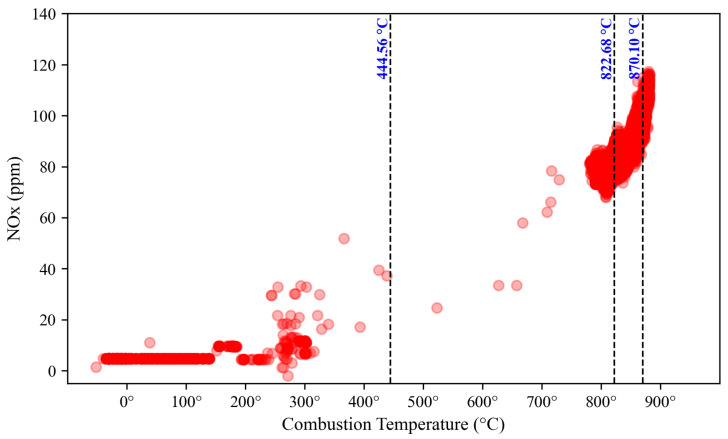
DLE gas turbine’s operating region based on NO_x_ emission clustering.

**Figure 19:**
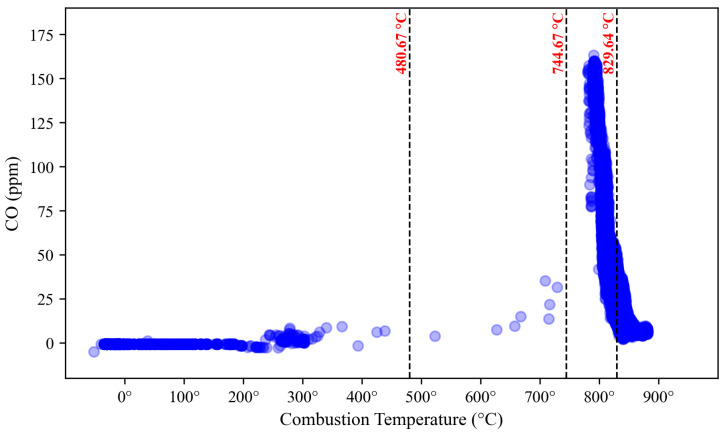
DLE gas turbine’s operating region based on CO emission clustering.

The prediction of the operating range is performed separately for NO_x_ and CO emissions, as depicted in Figure 18 and Figure 19, where the NO_x_ and CO data scattered with red and blue color, respectively. The dashed black vertical line represents the margin between two clusters calculated by averaging the distance of the nearest data point to one another. This margin cut the x-axis, the combustion temperature, to identify the range of each region. Based on the NO_x_ perspective, Region 1 is found below 444.56 °C, Region 2 ranges from 444.57 °C to 822.68 °C, Region 3 starts from 822.69 °C to 870.10 °C, and above 870.10 °C is labelled as Region 4. From the CO perspective, Region 1 is located below 480.67 °C, Region 2 ranges from 480.68 °C to 744.67 °C, Region 3 is found at 744.68 °C to 829.64 °C, and Region 4 is located above 829.64 °C.

**Figure 20:**
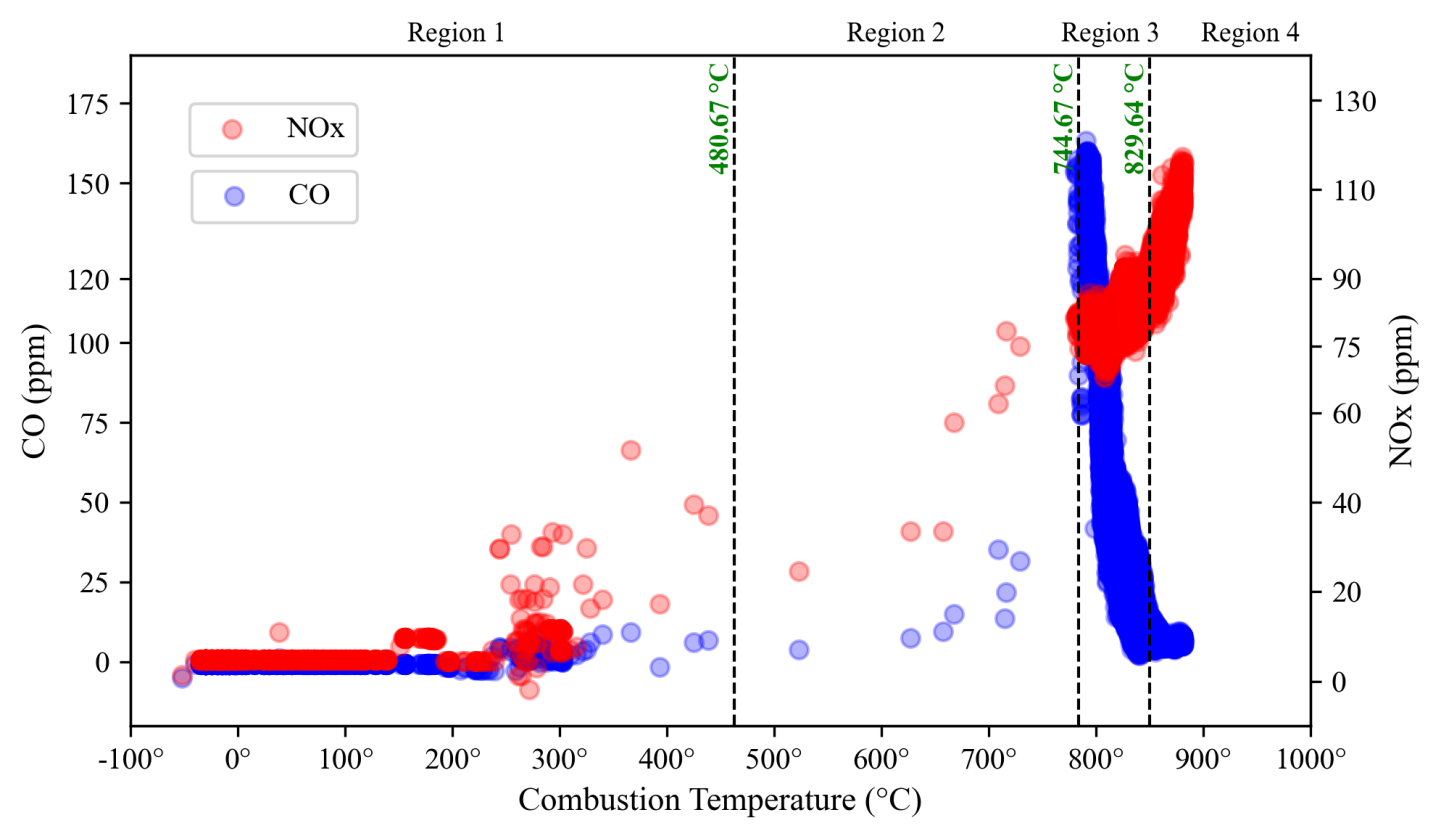
Finalized operating region of the DLE gas turbine.

The regions identified from NO_x_ and CO clustering are subsequently used to determine the final range by averaging the margin from both sides to find the optimum range, as tabulated in Table 5 and depicted in Figure 20. Each region portrays different operation conditions of DLE gas turbines which are assigned as trip, near to trip, safe operation, and unhealthy.

As a tight control of DLE gas turbine operation requires a specific range to operate safely, the proposed model predicts the optimum range, which starts from 744.68 °C to 829.64 °C (Region 3), as shown in Figure 20. The operation at 480.68 °C to 744.67 °C (Region 2) is considered near the trip, indicating a high tripping probability, which can be used as a prevention alarm to avoid tripping issues. Hence, the operator can act accordingly by controlling the system to restore the turbine to normal operation. This action also can be referred to prevent the LBO fault and high formation of CO emission. The turbine may experience a trip when operated below 480.67 °C (Region 1). In contrast, an unhealthy operation may occur when the turbine is operated above 829.64 °C, leading to a high formation of NO_x_ emissions.

## 5. Conclusions

This paper presents a technique to predict the DLE gas turbine’s operating range using a semi-supervised approach. The prediction model is developed by hybridizing XGBoost and K-Means algorithms using an actual DLE gas turbine data with rated power of 17.9 MW. 15 parameters including operational and emissions concentration parameter are examined. Based on the correlation analysis, the important features which will be used for model developments are compressor discharge pressure, splitter opening, fuel gas flow, exhaust gas pressure, exhaust temperature, load, and ambient air temperature.

The XGBoost model predicts the turbine’s combustion temperature, NO_x_, and CO emissions. Then their predicted output is fed to the K-Means model for operating region prediction. Based on the result, it can be concluded that the XGBoost model successfully predicts the combustion temperature, NO_x_, and CO with the accuracy represented by $R^{2}$ of 0.9999, 0.9309, and 0.7109, respectively. Furthermore, the relative error percentage of these predicted outputs is lesser than 1%, which meets the decision criteria as requested by industry needs. Additionally, the proposed model outperforms other regression algorithms such as decision tree, linear regression, multilayer perceptron, and support vector machine. Based on the comparison between the mentioned algorithms, the decision tree model produced prediction results with high deviation, as observed on the graphical plot of the predicted and actual values for each output parameter. On the other hand, the proposed model exhibits an excellent prediction result in both numerical and graphical evaluation.

For operating region prediction, the optimal number of clusters is 4, representing the region of safe operation, unhealthy, near to trip, and trip zone. Based on the clustering result, the optimum operating range is found at 744.68 °C to 829.64 °C. The operation exceeding that range will lead the turbine to the unhealthy condition indicated by high production of NO emissions. On the other hand, the operation below that region will turn the turbine into a near-trip zone and finally lead to tripping issues. Further, it can cause a high formation of CO emissions. The advantages and drawbacks found from the analyzed algorithms are tabulated in Table 6.

The proposed model is expected to help the industry stakeholders and operators make the proper decision for more reliable operation of the DLE gas turbine while mitigating the tripping issues and maintaining low emissions production. Hence, the technique can be used as guidance for better load management and tripping prevention strategy, which is applicable to DLE gas turbines and other applications involving operating range prediction.

## Figures and Tables

**Table 1:** Hyperparametes for the proposed XGBoost model.

Hyperparameters	Combustion Temperature	NO_x_ Emission	CO Emission
max_depth	6	4	6
alpha	1.88	2.88	1
min_child_weight	1	0	1
reg_lambda	1	0.03	0.59
gamma	1.95	3.59	3.86
subsample	0.86	0.42	0.78
colsample_bytree	1	0.79	0.40
n_estimator	500	100	100
learning_rate	0.077	0.415	0.054

**Table 2:** DLE gas turbine’s parameters collected from the actual plant.

No.	Parameters	Abr.	Unit	Min.	Max.	Mean
1	Load	Load	MW	−0.10	18.31	8.66
2	Speed	Speed	RPM	−0.0005	5133.29	4583
3	Ambient Air Temperature	AAT	°C	25.17	41.90	31.87
4	Inlet Guide Vane Opening	IGVO	%	41.26	85.11	54.37
5	Compressor Discharge Pressure	CDP	kPag	−0.03	7.49	5.15
6	Stop Ratio Valve Opening	SRVO	%	−0.37	36.90	22.76
7	Gas Control Valve Opening	GCVO	%	0	49.12	30.43
8	Splitter Opening	SO	%	0	101.92	34.06
9	Fuel Gas Flow	FGF	kg/h	−1.31	142.13	86.48
10	Fuel Gas Pressure	FGP	kPag	−0.003	16.31	14.34
11	T5 Combustion Temperature	CT	°C	−77.94	966.27	742.89
12	T7 Exhaust Temperature	ET	°C	0	531.73	428.29
13	Exhaust Gas Pressure	EGP	kPag	−0.55	8.31	2.99
14	NO_x_	NO_x_	ppm	0	137.69	75.04
15	CO	CO	ppm	0	203.86	26.98

**Table 3:** Correlation of input and output parameters.

Variable	CT	NO_x_	CO
Load	0.98	0.98	0.091
Speed	0.99	0.96	0.26
AAT	−0.65	−0.61	−0.21
IGVO	0.99	0.95	0.25
CDP	1	0.97	0.23
SRVO	0.99	0.97	0.19
GCVO	0.99	0.97	0.2
SO	−0.99	−0.97	−0.24
FGF	0.99	0.97	0.19
FGP	0.99	0.96	0.26
ET	1	0.97	0.2
EGP	0.97	0.95	0.16

**Table 4:** Performance evaluation of regression model.

Output	Performance	DT	LR	MLP	SVM	XGBoost
Combustion Temperature	$R^{2}$	0.9979	0.9996	0.9993	0.9986	**0.9999**
	MAE	1.3321	0.8624	1.2608	0.9227	**1.1285**
	RMSE	7.3604	3.1384	4.4781	6.0692	**6.9549**
	% error	0.0244	0.0037	0.0856	0.0053	**0.0356**
NO_x_ emission	$R^{2}$	0.9053	0.8008	0.9272	0.9211	**0.9309**
	MAE	4.1907	4.5393	3.2349	3.9201	**3.5968**
	RMSE	5.8276	8.7526	5.0762	5.6715	**4.9765**
	% error	7.2016	1.5261	1.1945	1.0638	**0.1168**
CO emission	$R^{2}$	0.6687	0.4076	0.6918	0.4604	**0.7109**
	MAE	15.3233	17.4969	14.1294	17.8439	**14.2486**
	RMSE	24.9892	34.6808	24.8311	33.0383	**23.7482**
	% error	14.0473	30.7938	20.2803	20.0774	**0.9200**

**Table 5:** Finalized operating range of the DLE gas turbine.

Region	Operating Range	Description
1	<480.67 °C	Trip
2	480.68 °C–744.67 °C	Near to Trip
3	744.68 °C–829.64 °C	Safe Operation
4	>829.64 °C	Unhealthy

**Table 6:** Summary of the advantages and disadvantages of all algorithms.

Algorithms	Mode	Advantages	Disadvantages
XGBoost	Supervised	• Less time for model training • Excellent dealing with missing data and outliers • Few hyperparameters for model tuning	• Limited to supervised learning application, which is unable to capture the ambiguity in operating range prediction
Mulitlayer Perceptron	Supervised	• Has a self-learning function • Adaptive either for large or small data set with relatively same accuracy	• Computationally complex • Cannot perform well when data is insufficient
Support Vector Machine	Supervised	• Able to produce a model with low variance • Relatively insensitive to overfitting	• Time-consuming due to too many parameters connected • Sensitive to noisy data and outliers
Decision Tree	Supervised	• Computationally efficient and handle large datasets easily • Robust to outliers and noise in the data	• Struggle with missing data • Produce relatively high deviation in emission prediction
Linear Regression	Supervised	• Simple and easy to implement • Has a good generalizability to predict new data	• Limited to linear relationship between independent and dependent variables • May suffer from over-fitting when the model becomes too complex
K-Means	Unsupervised	• Excellent in clustering that can be used in range prediction cases • Good dealing with uneven distributed data • Able to handle huge data	• Bad in handling noisy data and outliers.

## Data Availability

Not applicable.
